# Asymptomatic oral yeast carriage and antifungal susceptibility profile of HIV-infected patients in Kunming, Yunnan Province of China

**DOI:** 10.1186/1471-2334-13-46

**Published:** 2013-01-28

**Authors:** Yu-Ye Li, Wen-Ying Chen, Xia Li, Hong-Bin Li, Hui-Qin Li, Li Wang, Li He, Xin-Ping Yang, Xi-Cheng Wang, Yun-Li Huang, Yong-Gang Yao

**Affiliations:** 1 Department of Dermatology and Venereology, The First Affiliated Hospital of Kunming Medical University, Kunming, Yunnan, 650032, China; 2 Yunnan Provincial Hospital of Infectious Disease/AIDS Care Center (YNACC), Anning, Yunnan, 650300, China; 3 Key Laboratory of Animal Models and Human Disease Mechanisms of Chinese Academy of Sciences & Yunnan Province, Kunming Institute of Zoology, Kunming, Yunnan, 650223, China; 4 Yunnan Institute of Dermatology and Venereology, Kunming, Yunnan, 650032, China

**Keywords:** Oral yeast, Colonization, Antifungal susceptibility, HIV, Chinese

## Abstract

**Background:**

Oral *Candida* colonization and its relation with predisposing factors in HIV-infected patients have received wide concerns during recent decades. In this study, we investigated asymptomatic oral *Candida* carriage rate, species distribution and antifungal susceptibility of 604 HIV-infected patients and 851 healthy individuals in Kunming, Yunnan Province of China.

**Methods:**

Mucosal swab sampling was taken from each subject and CHROMagar *Candida* agar medium and API 20C AUX system were used to identify yeast isolates. *In vitro* antifungal susceptibility was tested by the broth microdilution method according to the M27-A2 document of the Clinical and Laboratory Standard Institute (CLSI).

**Results:**

The oral yeast colonization rate in HIV-infected patients (49.5%) was higher than that of healthy subjects (20.7%). *Candida albicans* constituted the most frequent species, accounting for 82.2% of yeast isolates. The remaining species were composed of *C. glabrata*, *C. parapsilosis*, *C. krusei*, *C. tropicalis*, *C. rugosa*, *C. norvegensis*, *Pichia ohmeri* and *Saccharomyces cerevisiae*. In HIV-infected patients, asymptomatic oral yeast colonization was associated with low CD4 cell count (<200 cells/mm^3^) and lack of highly active antiretroviral therapy (HAART). Different *Candida* species isolated from our samples presented different susceptibility to voriconazole, fluconazole and itraconazole. Amphotericin B had the best inhibiting effect for all isolates.

**Conclusion:**

Oral yeast colonization in Han Chinese patients with HIV from Kunming had common and unique features and was associated with CD4 cell number and HARRT. Amphotericin B should be used with first priority in controlling *Candida* infection in Han Chinese patients from Kunming. Our results provide first hand information on monitoring oral yeasts colonization in HIV-infected patients from Kunming, China.

## Background

It is estimated by the Chinese Ministry of Health and the Joint United Nations Programme on HIV/AIDS (UNAIDS) that at the end of 2009, there were 740,000 living people infected with human immunodeficiency virus (HIV) in China, including 105,000 patients suffering acquired immunodeficiency syndrome (AIDS) [1]. Among these HIV/AIDS endemic areas, Yunnan Province is heavily affected and has the longest history of infection in China, simply because of diverse ethnic composition and bordering with the “Golden triangle” of illicit opium production [2]. Despite of remarkable evolution of China’s response to HIV/AIDS [3] and achievements regarding the coverage on highly active antiretroviral therapy (HARRT) and the decrease of HIV-related mortality, urgent needs are required for early HIV diagnosis, better access to HARRT and medical supervision of patients with HIV/AIDS [4].

*Candida* species are composed of commensal fungal organisms and opportunistic pathogens of mucosal tissues [5]. These species can lead to a variety of mucosal infections under the imbalance of oral microflora or suffering with systemic diseases. Human resistance to *Candida* was affected by innate and acquired immunity [5]. In HIV-infected patients, there are many reports for a high incidence of asymptomatic oral yeast carriage [6-11]. In particular, oropharyngeal candidiasis (OPC) is the most frequent opportunistic infection, with a occurrence rate in 50% to 90% of patients during disease progression [12]. Presence of asymptomatic carriage of yeasts in the oral cavities of HIV-infected patients and lower CD4 cell count have been associated with subsequent development of OPC [6,13]. Among *Candida* species isolated from the oral cavity, *Candida albicans* is the most prevalent species; there are many other yeast species characterized by low susceptibility to azole, such as *C. glabrata*, *C. krusei*, *C. tropicalis*, *C. parapsilosis*[14,15]. Previous study have shown that HAART decreased the incidence of all HIV-related opportunistic infections including fungal infection [16], but there are conflicting reports regarding the association between oral yeast colonization and HAART [6,7,17-19]. Monitoring the antifungal susceptibility of clinical isolates from patients with HIV/AIDS has crucial importance for better treatment of these patients [20].

Hitherto, large-scale investigations have not been sufficiently performed to characterize the oral fungal carriage rate, species prevalence and antifungal susceptibility among HIV-infected individuals in China. In this study, we analyzed 604 HIV-infected patients and 851 healthy individuals in Kunming, Yunnan Province, China, with an intention to (1) determine the carriage rate and species distribution in HIV-infected patients and healthy individuals from Kunming, (2) to investigate the risk factors associated with oral yeast colonization among HIV-infected patients, and (3) to evaluate antifungal susceptibility profile of oral fungal isolates from Han Chinese patients with HIV/AIDS.

## Methods

### Patients

HIV-infected patients from Yunnan Provincial Hospital of Infectious Disease/AIDS Care Center (YNACC) were recruited in this study from June 2009 to November 2009. Patients were eligible for the study if they received no treatment with antifungal or antimicrobial agents during the previous 6 months before sampling and had no sign for clinical evidence of OPC according to the criteria of the EC-Clearinghouse on oral problems related to HIV infection [21]. A total of 604 patients from Kunming met the criteria and were analyzed in this study. Clinical and demographic data taken at the time of oral examination for each patient included the following items: age, gender, marriage, mode of HIV transmission, CD4 cell count, and HAART. Simply for convenience and for comparison with the HIV-infected patients, we randomly collected 851 healthy donors from Kunming who attended routine physical examinations at the First Affiliated Hospital of Kunming Medical University and were confirmed to be HIV-negative and without any clinical sign for OPC. Informed consents conforming to the tenets of the Declaration of Helsinki were obtained from each participant prior to this study. The institutional review board of the First Affiliated Hospital of Kunming Medical University approved this study.

### Sample collection and culture

Samples were obtained by swabbing buccal mucosa and tongue with a sterile cotton swab, then were plated onto Sabouraud’s dextrose agar (SDA) (bioMérieux, France) and incubated at 37°C for 48 h. Yeast colonies growing on each SDA tube were resuspended and 10 μL of suspension solution was used to inoculate plates with CHROMagar *Candida* agar medium (CHROMagar Company, France), followed by a two-day culture at 37°C [22]. We tentatively classified yeast species according to the colour of each colony: green colonies were identified as *C. albicans* or *C. dubliniensis*; dark green colonies were grouped as *C. dubliniensis*; colonies with blue, pink and mauve colours were regarded as *C. tropicalis*, *C. krusei* and *C. glabrata*[23]. Isolates of *C. albicans* were further studied by the germ-tube test and chlamydospore production. Colonies of non-*C. albicans* species were subcultured on new SDA medium, and species identifications were validated by API 20C AUX system for yeasts (bioMérieux, France). Discrimination between *C. albicans* and *C. dubliniensis* was investigated by analyses of germ tube formation in calf serum at 37°C for 3 h, degree of chlamydospore production on cornmeal agar supplemented with 1% tween-80 growth at 45°C on SDA, colony morphology at Staib agar, and xylose assimilation test.

### Antifungal susceptibility testing

*In vitro* antifungal susceptibility was tested by the broth microdilution method according to the M 27-A2 document of the Clinical and Laboratory Standard Institute (CLSI) [24]. Four antifungal agents (amphotericin B, itraconazole, fluconazole and voriconazole) obtained from Sigma (USA) were used. These four drugs were widely used in our clinical center to cure patients with fungal infection. The culture medium was RPMI-1640 with L-glutamine but no bicarbonate, and buffered at pH 7.0 with 0.165 mol/L MOPS. The range of drug concentration was 0.125-64 μg/mL for fluconazole and 0.031-16 μg/mL for all three drugs (amphotericin B, itraconazole and voriconazole). Cell density of inoculum suspension was adjusted with a spectrophotometer and was diluted to achieve a final concentration of 1 × 10^3^ to 5 × 10^3^ cells / mL by RPMI-1640. The microplates were incubated at 35°C and end point read at 48 h. The minimal inhibitory concentration (MIC) of amphotericin B was defined as the lowest concentration that prevents any discernible growth. The MIC for azole was defined as the lowest concentration that sharply decreases the growth relative to the control, as it is not possible to distinguish the difference between 50% and 80% inhibition by eye when using the microdilution method. Susceptible (S) breakpoints were set as MIC ≤8 μg/mL for fluconazole, ≤0.125 μg/mL for itraconazole, <1 μg/mL for voriconazole and ≤ 1 μg/mL for amphotericin B, respectively. Species with MICs between 16 μg/mL and 32 μg/mL for fluconazole and between 0.25 μg/mL and 0.5 μg/mL for itraconazole were considered as susceptible–dose dependent (S-DD). Species with MICs ≥64 μg/mL for fluconazole, ≥1 μg/mL for itraconazole and voriconazole, and >1 μg/mL for amphotericin B were considered as resistant (R) [8,24]. Quality control was performed by testing the reference strains of *C. parapsilosis* ATCC 22019 and *C. krusei* ATCC 6258.

### Data analysis

Statistical analysis was performed by SPSS Version 16.0. Differences of gender, marriage, mode of HIV transmission and HAART were assessed by the Chi-squared test between the HIV infected patients with and without oral yeast colonization. Differences of age and CD4 cell count were evaluated by the Mann–Whitney test as these variables were non-normally distributed in our patients. We also conducted binary logistic regression analysis to determine whether differences in cases and controls remain after adjustment for socio-demographic and clinical characteristics. A *P* value of <0.05 was considered as statistically significant.

## Results

The patients with HIV (230 female and 374 male) had a median age of 37.7 years old (range from 18 to 75 years old). Among these patients, around 45.2% (273/604) was infected through heterosexual contact, while 26.5% of them (160/604) was injecting drug user (IDU), followed by homosexual male (22/604 = 3.6%). Only five patients (0.8%) were affected via transfusion and one patient (0.2%) was a blood and plasma donor. The remaining 143 patients (23.7%) had self-reported an unclear history of HIV infection. The average CD4 cell count was 253.4 cells/mm^3^, with a range from 2 to 1050 cells/mm^3^ in all patients. Most of patients (502/604 = 83.1%) were treated with HAART. Of the treated subjects, antiretroviral regimen of 481 patients (95.8%) was 2 NRTIs (nucleoside reverse transcriptase inhibitors) + 1 NNRTI (non-nucleoside reverse transcriptase inhibitor). Only 21 patients (4.2%) received 2NRTIs + 1PI (protease inhibitor). The control group consisted of 411 male and 440 female healthy donors, with a mean age of 39.3 years old (range from 18 to 74 years old).

Oral yeast carriage rates were 49.5% (299/604) in HIV-infected patients and 20.7% (176/851) in healthy controls, and the difference was statistically significant (Chi-square test, *P* < 0.001). The distribution of fungal species among oral isolates collected from HIV-infected subjects and controls were shown in Table 1. In HIV-infected patients, *C. albicans* accounted for 82.2% of the recognized species. We also identified *C. glabrata*, *C. parapsilosis*, *C. krusei*, *C. tropicalis*, *C. rugosa*, *C. norvegensis*, *Pichia ohmeri* and *Saccharomyces cerevisiae*, albeit at different frequencies. *C. albicans* was also the prevalent isolated species (73.2%) in the control group, but the frequency was significantly lower than that of HIV-infected patients (*P* = 0.022). We identified *C. lusitaniae* in one healthy donor and *C. guilliermondii* in another healthy donor but not in any HIV-infected patients (Table 1). There are several cases with multiple species isolation among oral isolates collected from both the patients (21/299) and controls (3/176), despite the fact that this is more common in patients with HIV (Additional file 1).

**Table 1:** Distribution of oral yeast isolates in 604 HIV-infected patients and 851 healthy subjects from Kunming, Yunnan Province of China

**Species**	**HIV-infected patients**		**Healthy subjects**^**a**^	
	**Occurrence of species (%)**^**a**^	**Proportion of HIV-infected patients with colonization**^**b**^	**Occurrence of species (%)**^**a**^	**Proportion of HIV-infected patients with colonization**^**b**^
*C. albicans*	263 (82.2)	43.5	131 (73.2)	15.4
*C. glabrata*	29 (9.1)	4.8	17 (9.5)	2.0
*C. parapsilosis*	12 (3.8)	2.0	21 (11.7)	2.5
*C. krusei*	10 (3.1)	1.7	3 (1.7)	0.4
*C. tropicalis*	2 (0.6)	0.3	4 (2.2)	0.5
*C. rugosa*	1 (0.3)	0.2	0 (0.0)	0.0
*C. norvegensis*	1 (0.3)	0.2	0 (0.0)	0.0
*C. lusitaniae*	0 (0.0)	0.0	1 (0.6)	0.1
*C. guilliermondii*	0 (0.0)	0.0	1 (0.6)	0.1
*Pichia ohmeri*	1 (0.3)	0.2	1 (0.6)	0.1
*Saccharomyces cerevisiae*	1 (0.3)	0.2	0 (0.0)	0.0
Total	320 (100.0)	−	179 (100.0)	−

^a^ The values in parentheses refer to frequency of each species in all oral yeast isolates.

^b^ Multiple species isolation was counted according to the presence of each species in a donor.

We performed association analysis to discern predisposing factors for oral fungal colonization. There are significant differences in CD4 cell count (Mann-Whithney test, *P* = 0.019) and HAART (Chi-square test, *P* = 0.00002) between patients with and without oral fungal colonization. However, carriage of oral yeast was not associated with mean age (Mann-Whithney test, *P* = 0.837), gender (Chi-square test, *P* = 0.981), marriage (Chi-square test, *P* = 0.703), and HIV transmission (Chi-square test, *P* = 0.351). There was no difference regarding the frequency of oral yeast colonization between the two group of patients treated with different antiretroviral regimens (Chi-square test, *P* = 0.851) (Table 2). Similar results were obtained when we conducted the adjusted analysis using the binary logistic regression modeling to determine whether differences in cases and controls remain after adjustment for socio-demographic and clinical characteristics (Additional file 2).

**Table 2:** Association of oral yeast colonization with demographic and clinical features in 604 HIV-infected patients from Kunming, Yunnan Province of China

**Parameter**		**Colonized**	**Non-colonized**	***P*-value**
		**n = 299 (%)**	**n = 305 (%)**	
Mean age (years)		38.0 ± 10.8	37.5 ± 9.2	0.837 ^a^
Gender (female)		114 (38.1)	116 (38.0)	0.981^b^
Marriage	Unmarried	79 (26.4)	79 (25.9)	0.703
	Married	202 (67.6)	200 (65.6)	
	Divorced	14 (4.7)	20 (6.6)	
	Widowhood	4 (1.3)	6 (2.0)	
Transmission	Injecting drug user	83 (27.8)	77 (25.2)	0.605
	Heterosexual sex	131 (43.8)	142 (46.6)	
	Homo/bisexual male	8 (2.7)	14 (4.6)	
	Transfusion recipient	3 (1.0)	2 (0.7)	
	Blood and plasma donors	0 (0.0)	1 (0.3)	
	Unknown	74 (24.7)	69 (22.6)	
CD4 cells/mm^3^	<200	134 (44.8)	103 (33.8)	0.019
	200-500	141 (47.2)	176 (57.7)	
	>500	24 (8.0)	26 (8.5)	
HAART		229 (76.6)	273 (89.5)	0.00002
Antiretroviral regimen ^c^	2NRTIs + 1NNRTI	219 (95.6)	262 (96.0)	0.851
	2NRTIs + 1PI	10 (4.4)	11 (4.0)	

^a^ Mann-Whithney test.

^b^ Chi-square test.

^c^ NRTIs, nucleoside reverse transcriptase inhibitors; NNRTI, non-nucleoside reverse transcriptase inhibitor; PI, protease inhibitor.

The MIC range, number of S strains, number of S-DD strains, and number of R strains to fluconazole, itraconazole, voriconazole and amphotericin B for oral yeast isolates from HIV-infected patients and healthy subjects were listed in Tables 3 and 4, respectively. Eighteen isolates (5.6%) were resistant to fluconazole, 44 (13.8%) were resistant to itraconazole, and 17 (5.3%) were resistant to voriconazole. All of the isolates were susceptible to amphotericin B (Table 3). Among those healthy subjects, 5 (2.8%) were resistant to fluconazole, 23 (12.8%) were resistant to itraconazole, and 4 (2.2%) were resistant to voriconazole. None of the isolates from healthy donors were resistant to amphotericin B (Table 4).

**Table 3:** Antifungal susceptibility profile of 320 oral yeast isolates from HIV-infected patients from Kunming, China

**Species (No.)**	**Antifungal agent**	**MIC range (μg/mL)**	**No. of S strains (%)**^**a**^	**No. of S-DD strains (%)**^**b**^	**No. of R strains (%)**^**c**^
*C. albicans* (263)	Fluconazole	0.125-64	251 (95.0)	3 (1.1)	9 (3.4)
	Itraconazole	0.031-16	232 (88.2)	13 (4.9)	18 (6.8)
	Voriconazole	0.031-16	255 (97.0)	-	8 (3.0)
	Amphotericin B	0.125-1	263 (100.0)	-	0 (0)
*C. glabrata* (29)	Fluconazole	0.25-64	23 (79.3)	1 (3.4)	5 (17.2)
	Itraconazole	0.031-16	7 (24.1)	7 (24.1)	15 (51.7)
	Voriconazole	0.031-16	25 (86.2)	-	4 (13.8)
	Amphotericin B	0.25-1	29 (100.0)	-	0 (0)
*C. krusei* (10)	Fluconazole	8-64	3 (30.0)	5 (50.0)	2 (20.0)
	Itraconazole	0.125-4	1 (10.0)	3 (30.0)	6 (60.0)
	Voriconazole	0.063-2	8 (80.0)	-	2 (20.0)
	Amphotericin B	0.25-1	10 (100.0)	-	0 (0)
*C. tropicalis* (2)	Fluconazole	64	0 (0)	0 (0)	2 (100)
	Itraconazole	16	0 (0)	0 (0)	2 (100)
	Voriconazole	16	0 (0)	-	2 (100)
	Amphotericin B	0.5-1	2 (100.0)	-	0 (0)
*C. parapsilosis* (12)	Fluconazole	0.125-1	12 (100.0)	0 (0)	0 (0)
	Itraconazole	0.031-2	6 (50.0)	4 (33.3)	2 (16.7)
	Voriconazole	0.031	12 (100.0)	-	0 (0)
	Amphotericin B	0.25-1	12 (100.0)	-	0 (0)
*C. rugosa* (1)	Fluconazole	1	1 (100.0)	0 (0)	0 (0)
	Itraconazole	0.031	1 (100.0)	0 (0)	0 (0)
	Voriconazole	0.031	1 (100.0)	-	0 (0)
	Amphotericin B	0.5	1 (100.0)	-	0 (0)
*C. norvegensis* (1)	Fluconazole	1	1 (100.0)	0 (0)	0 (0)
	Itraconazole	0.25	1 (100.0)	0 (0)	0 (0)
	Voriconazole	0.063	1 (100.0)	-	0 (0)
	Amphotericin B	1	1 (100.0)	-	0 (0)
*Pichia ohmeri* (1)	Fluconazole	8	1 (100.0)	0 (0)	0 (0)
	Itraconazole	2	0 (0)	0 (0)	1 (100)
	Voriconazole	0.125	1 (100.0)	-	0 (0)
	Amphotericin B	0.5	1 (100.0)	-	0 (0)
*Saccharomyces cerevisiae* (1)	Fluconazole	16	0 (0)	1 (100.0)	0 (0)
	Itraconazole	0.25	0 (0)	1 (100.0)	0 (0)
	Voriconazole	0.125	1 (100.0)	-	0 (0)
	Amphotericin B	1	1 (100.0)	-	0 (0)

^a^ Susceptible (S) strains were defined by MIC ≤8 μg/mL for fluconazole, ≤0.125 μg/mL for itraconazole, <1 μg/mL for voriconazole and ≤ 1 μg/mL for amphotericin B.

^b^ Susceptible-dose dependent (S-DD) strains were defined by MICs between 16 μg/mL and 32 μg/mL for fluconazole and between 0.25 μg/mL and 0.5 μg/mL for itraconazole.

^c^ Resistant (R) strains were defined by MIC ≥64 μg/mL for fluconazole, ≥1 μg/mL for itraconazole and voriconazole, and >1 μg/mL for amphotericin B.

**Table 4:** Antifungal susceptibility profile of 179 oral yeast isolates from 851 healthy subjects from Kunming, China

**Species (No.)**	**Antifungal agent**	**MIC range (μg/mL)**	**No. of S strains (%)**^**a**^	**No. of S-DD strains (%)**^b^	**No. of R strains (%)**^**c**^
*C. albicans* (131)	Fluconazole	0.125-64	127 (96.9)	2 (1.5)	2 (1.5)
	Itraconazole	0.031-16	121 (92.4)	2 (1.5)	8 (6.1)
	Voriconazole	0.031-8	129 (98.5)	-	2 (1.5)
	Amphotericin B	0.125-1	131 (100.0)	-	0 (0)
*C. glabrata* (17)	Fluconazole	0.25-64	15 (88.2)	0 (0)	2 (11.8)
	Itraconazole	0.031-16	6 (35.3)	0 (0)	11 (64.7)
	Voriconazole	0.031-8	16 (94.1)		1 (5.9)
	Amphotericin B	0.5-1	17 (100.0)	-	0 (0)
*C. krusei* (3)	Fluconazole	4-64	2 (66.7)	0 (0)	1 (33.3)
	Itraconazole	1-2	0 (0)	0 (0)	3 (100)
	Voriconazole	0.125-2	2 (66.7)	-	1 (33.3)
	Amphotericin B	0.5-1	3 (100.0)	-	0 (0)
*C. tropicalis* (4)	Fluconazole	0.25-4	4 (100.0)	0 (0)	0 (0)
	Itraconazole	0.031-0.25	2 (50.0)	2 (50.0)	0 (0)
	Voriconazole	0.031-0.063	4 (100.0)	-	0 (0)
	Amphotericin B	0.5-1	4 (100.0)	-	0 (0)
*C. parapsilosis* (21)	Fluconazole	0.125-2	21 (100.0)	0 (0)	0 (0)
	Itraconazole	0.031-0.25	13 (61.9)	8 (38.1)	0 (0)
	Voriconazole	0.031-0.25	21 (100.0)	-	0 (0)
	Amphotericin B	0.25-1	21 (100.0)	-	0 (0)
*C. lusitaniae* (1)	Fluconazole	0.25	1 (100.0)	0 (0)	0 (0)
	Itraconazole	0.031	1 (100.0)	0 (0)	0 (0)
	Voriconazole	0.031	1 (100.0)	-	0 (0)
	Amphotericin B	1	1 (100.0)	-	0 (0)
*C. guilliermondii* (1)	Fluconazole	4	1 (100.0)	0 (0)	0 (0)
	Itraconazole	0.5	0 (0)	1 (100.0)	0 (0)
	Voriconazole	0.063	1 (100.0)	-	0 (0)
	Amphotericin B	1	1 (100.0)	-	0 (0)
*Pichia ohmeri* (1)	Fluconazole	2	1 (100.0)	0 (0)	0 (0)
	Itraconazole	2	0 (0)	0 (0)	1 (100)
	Voriconazole	0.063	1 (100.0)	-	0 (0)
	Amphotericin B	1	1 (100.0)	-	0 (0)

^a^ Susceptible (S) strains were defined by MIC ≤8 μg/mL for fluconazole, ≤0.125 μg/mL for itraconazole, <1 μg/mL for voriconazole and ≤ 1 μg/mL for amphotericin B.

^b^ Susceptible-dose dependent (S-DD) strains were defined by MICs between 16 μg/mL and 32 μg/mL for fluconazole and between 0.25 μg/mL and 0.5 μg/mL for itraconazole.

^c^ Resistant (R) strains were defined by MIC ≥64 μg/mL for fluconazole, ≥1 μg/mL for itraconazole and voriconazole, and >1 μg/mL for amphotericin B.

## Discussion

### Oral yeast colonization in Han Chinese patients with HIV from Kunming had common and unique features

In this study, we screened oral fungal colonization in the largest cohort of Han Chinese patients with HIV from Kunming, Yunnan Province to date. This province has been heavily hit by HIV infection [2,3] but similar research has rarely been performed before. As oral yeast colonization can be affected by different factors, especially oral hygiene, we only analyzed patients with HIV and healthy controls from Kunming to eliminate potential influence of geographic region and oral hygiene habits of urban and rural residents. The overall pattern of oral yeast colonization in Chinese patients from Kunming was similar to those of previously described in other populations [6,7,17], but also presented some population- and/or region- unique features. The reported oral yeast carriage rate of HIV-infected patients varied in different populations across world, mainly due to the different approaches for yeast sampling and geographic and/or ethnic differences [25]. The colonization rate (49.5%) in Han Chinese patients infected with HIV from Kunming was similar to that of Brazilian [11,19], but lower than that of South Indian [9,26] and higher than that of Han Chinese in Beijing, China [27]. Most of previous studies showed a higher prevalence of asymptomatic oral yeast carriage in HIV-infected patients than in healthy group [6,7,17] and this pattern was also confirmed in the current study.

Among the yeast species identified in Han Chinese patients with HIV from Kunming, *Candida albicans* was the predominant one (82.2%). This observation was consistent with previous observations for Tanzanian patients [10] and Brazilian patients [19]. A total of 57 (17.8%) non-*C. albicans* yeast strains that belonged to eight species were found in our HIV-infected patients, but the frequency of non-*C. albicans* yeast strains in our colonized samples was much lower than that of colonized patients in Turkey (50.9%) [28] and in Brazil (50.0%) [8]. The exact reason for this discrepancy remains unknown. In addition, our result showed that the presence of certain non-*C. albicans* yeast species was not related to the status of HIV infection in Han Chinese from Kunming, and a substantial proportion of healthy donors were also colonized with non-*C. albicans* yeast species. This observation gave indirect support that colonization of non-*C. albicans* yeast species was merely by chance. Previous studies have shown a trend towards the increase of non-*C. albicans* yeast species colonization in patients with HIV [8], and this trend had been elegantly demonstrated by a retrospective report in Chinese patients from Taiwan, China, in which non-*C. albicans* yeast species accounted for 9%, 14%, and 14.8% in years 1999, 2001, and 2002, respectively [16]. We were unable to perform such a test in this study as we only sampled these subjects once. Repeated exposure to antifungal agents and recurrent infections might account for the high colonization of the non-*C. albicans* species [14]. In contrast to the previous studies, we failed to find *C. dubliniensis*, which was initially isolated from HIV-infected patients with oropharyngeal candidosis [29] and HIV-negative individuals [7]. The absence of *C. dubliniensis* in our samples suggested for a restricted distribution of this species.

Co-colonization of multiple yeast species in oral cavity has been found in many studies [9,22,28]. In our study, 7% (21/299) of colonized HIV-infected patients and 1.7% (3/176) colonized healthy individuals had multiple yeast isolates. In particular, combination of *C. albicans* and *C. glabrata* was the predominant type. This observation is different from that of South Indian, in which colonization of both *C. albicans* and *C. krusei* was the most common and there was a colonization of three *Candida* species [9]. Among the three healthy individuals with colonization of multiple yeast species, one with *C. albicans* and *C. tropicalis* and two with *C. albicans* and *C. glabrata*, it would be interesting to have follow-up examinations for these individuals. The exact reason for the presence of *C. lusitaniae* and *C. guilliermondii* in healthy donors but not in any HIV-infected patients remains elusive.

For prospective identification of yeasts, additional application of updated methods, e.g. techniques which provide splitting up of *Candida parapsilosis*, *C. metapsilosis*, *C. orthopsilosis* and other related species, as well as to recognize potential new species will be employed in future studies. This would be of interest because there is a species specific difference in clinical relevance between *Candida* species, e.g. between *C. metapsilosis* and *C. orthopsilosis*[30]. Moreover, it would be rewarding to have a long-term surveillance program for monitoring the dynamics of the species in colonized HIV-infected patients and healthy individuals.

### Oral yeast colonization in Han Chinese patients infected with HIV was associated with CD4 cell number and HARRT

There was a debate over whether low CD4 cell count was associated with asymptomatic oral yeast carriage. In an early study, Fong et al. [6] found that the frequency of *C. albicans* carriage, the concentration of yeasts isolated and the development of thrush were all associated to CD4 cell count in patients from Canada. Delgado et al. [19] reported that Brazilian patients with CD4 cell count <200 cells/mm^3^ had significantly higher incidence of *Candida* in oral cavity. However, in another study based on 45 patients with HIV (23 female and 22 male), Back-Brito et al. [7] claimed that yeast carriage and *Candida* load were not associated with CD4 cell count; this negative observation might be caused by the relatively small number of patients analyzed. In this study, we analyzed 604 Han Chinese with HIV from Kunming and we thought our sample could help to solve this dispute. We found that asymptomatic oral yeast carriage was associated with a low CD4 cell count in our patients and CD4 cell count less than 200 cells/mm^3^ could be used as a predicted factor for yeast colonization (Table 2 and Additional file 2).

HAART has been said to strikingly decrease the rate of HIV-related opportunistic infections [16]. Absence of HAART increased colonization of *Candida spp.* in HIV-infected children from Brazil [17]. Antiretroviral protease inhibitors containing HIV aspartyl protease inhibitors could increase CD4 cell count [18] and inhibited the fungal secretory aspartyl proteinases, thus leading to reduced *Candida* infection during mucosal invasion [17]. In the present study, we found that patients without HAART had a higher incidence of *Candida* colonization, but oral yeast colonization had no association with the antiretroviral regimens including treatment with protease inhibitor. The exact reason underling these seemingly conflicting observations remains unknown. More samples undergoing protease inhibitor therapy should be recruited to answer this question in future study.

### *Candida* species in Han Chinese patients from Kunming had higher susceptibility to amphotericin B than to azole agents

Antifungal testing of oral yeast isolates are crucial for the selection of antifungal agents and the prediction of clinical response in patients with HIV/AIDS [20,26]. In this study, we tested the antifungal activities of four widely used drugs in Kunming on yeast isolates. We found that a relatively low percentage of *C. albicans* was resistant to voriconazole (3.0%), fluconazole (3.4%) and itraconazole (6.8%). The activity of voriconazole was better than the other two azole agents. However, a high azole-resistance rate was found for non-*C. albicans* yeast species, particularly for *C. glabrata*, *C. krusei* and *C. tropicalis*. All species were susceptible to polyene antifungal, amphotericin B, with a narrow range of MICs (Tables 3 and 4). This result clearly suggested that amphotericin B should be used with first priority in controlling *Candida* infection in Han Chinese patients from Kunming under certain circumstance. Although antifungal prophylaxis is not generally used in asymptomatic HIV positive patients, the antifungal susceptibility profile of oral yeast isolates obtained in this study will offer useful information for us to select proper drug to treat potential patients with OPC.

## Conclusions

By analyzing oral yeast colonization in 604 Han Chinese with HIV and 851 healthy donors from Kunming, Yunnan Province, we showed that HIV-infected patients had a higher *Candida* colonization rate than that of normal controls, which posed a higher risk for future symptomatic infection in HIV-infected patients. *C. albicans* was the predominant species for colonization in our samples. Asymptomatic oral yeast colonization was associated with low CD4 cell count (<200 cells/mm^3^) and the absence of HAART in HIV-infected patients. Different yeast isolates from Han Chinese with HIV presented different susceptibility to fluconazole, itraconazole, and voriconazole. Amphotericin B had the best inhibiting effect for all isolates. Our results provide first hand information on monitoring oral yeasts colonization in HIV-infected patients from Kunming, Yunnan, and will direct future medical treatment for these patients when they develop OPC. We will pursue further investigation to more extensively define the epidemiology and antifungal profiles of the *Candida* species in the future study.

## Competing interests

The authors declare no competing interests.

## Authors’ contributions

YYL, WYC and YGY designed the study. YYL and XCW coordinated the project. WYC, YYL, XL, HBL, HQL, LW, LH, XPY, XCW and YLH collected samples and clinical and experimental data. WYC, YYL, and YGY analyzed the data. WYC, YYL, and YGY drafted the manuscript. All authors read and approved the final manuscript.

## Pre-publication history

The pre-publication history for this paper can be accessed here:

http://www.biomedcentral.com/1471-2334/13/46/prepub

## Supplementary Material

Additional file 1 Multiple oral yeast species in HIV-infected patients and healthy subjects from Kunming, Yunnan Province of China. Click here for file

Additional file 2 Binary logistic regression analysis for the potential association of oral yeast colonization with demographic and clinical features in HIV-infected patients from Kunming, China. Click here for file
