# Supplementary material for: Asymptomatic oral yeast carriage and antifungal susceptibility profile of HIV-infected patients in Kunming, Yunnan Province of China
Source: BMC Infect Dis. 2013 Jan 28;13:46. doi: 10.1186/1471-2334-13-46 (PMC3641955; doi:10.1186/1471-2334-13-46)
Supplement: Additional file 1 — Multiple oral yeast species in HIV-infected patients and healthy subjects from Kunming, Yunnan Province of China. [file 1471-2334-13-46-S1.doc]

**Additional file 1: Multiple oral yeast species in HIV-infected patients and healthy subjects from Kunming, Yunnan Province of China**

| Species isolation | HIV-infected patient (n=604) | Healthy subject (n=851) |
| --- | --- | --- |
| *C. albicans + C. glabrata* | 15 | 2 |
| *C. albicans + C. krusei* | 3 | 0 |
| *C. albicans + C. tropicalis* | 0 | 1 |
| *C. albicans + C. rugosa* | 1 | 0 |
| *C. glabrata + C. parapsilosis* | 1 | 0 |
| *C. glabrata + Pichia ohmeri* | 1 | 0 |
| Total | 21 | 3 |
