# Supplementary material for: Asymptomatic oral yeast carriage and antifungal susceptibility profile of HIV-infected patients in Kunming, Yunnan Province of China
Source: BMC Infect Dis. 2013 Jan 28;13:46. doi: 10.1186/1471-2334-13-46 (PMC3641955; doi:10.1186/1471-2334-13-46)
Supplement: Additional file 2 — Binary logistic regression analysis for the potential association of oral yeast colonization with demographic and clinical features in HIV-infected patients from Kunming, China. [file 1471-2334-13-46-S2.doc]

**Additional file 2: Binary logistic regression analysis for the potential association of oral yeast colonization with demographic and clinical features in HIV-infected patients from Kunming, China**

| Factor | B | SE | *P* value | OR | 95% CI |
| --- | --- | --- | --- | --- | --- |
| Gender | -0.095 | 0.181 | 0.600 | 0.910 | 0.638-1.296 |
| Age | -0.002 | 0.009 | 0.846 | 0.998 | 0.981-1.016 |
| Marriage | 0.112 | 0.149 | 0.454 | 1.118 | 0.834-1.499 |
| Transmission | 0.096 | 0.077 | 0.212 | 1.101 | 0.947-1.280 |
| CD4 cells/mm^3^ | 0.001 | 0.001 | 0.041 | 1.001 | 1.000-1.002 |
| HAART | -0.765 | 0.248 | 0.002 | 0.465 | 0.286-0.757 |

B - Regression coefﬁcient; SE - Standard error; OR - Odds ratio; CI - Conﬁdence interval.
